# Extraintestinal Amoebiasis in Women after 6^th^ Day of Delivery

**DOI:** 10.1155/2021/1395404

**Published:** 2021-09-20

**Authors:** Awoke Minwuyelet, Yibeltal Aschale, Solomon Ayenew

**Affiliations:** ^1^Bichena Primary Hospital, Amhara Region, Ethiopia; ^2^Department of Medical Laboratory Science, College of Health Science, Debre Markos University, Debre Markos, Ethiopia

## Abstract

**Background:**

Extraintestinal amoebiasis is more common in countries with lower socioeconomic status. Complication related to amoebiasis is common in pregnant patients with malnutrition and others. Severe cases can be associated with high fatality rates. We would like to report a patient with a presumptive diagnosis of extraintestinal amoebiasis who was on the 6th postpartum day after intrauterine fetal death (IUFD). *Case Presentation*. The patient was a 31 year-old female who was on 6th postpartum day after IUFD after the 9th month of amenorrhea. She presented with severe epigastric pain, hiccups, and bloody vomiting of ingested matter for 5 days. She also had right upper quadrat pain and fatigue. In addition, she had generalized body weakness and yellowish discoloration of the eyes for one week. Moreover, she had pruritus, fever, and a history of watery diarrhea 6 days ago which got subsided during the presentation. Laboratory investigation indicated leukocytosis and increased level of alkaline phosphatase and direct and total bilirubin. Trophozoite of *E. histolytica* was seen on stool microscope, negative for viral marker and *Helicobacter pylori*. Abdominal ultrasonography showed normal liver parenchyma and biliary system. She was treated onsite with 500 mg metronidazole and 500 mg ceftriaxone for five days and discharged with metronidazole 500 mg PO three times a day and cloxacillin 500 mg PO four times a day for 7 days.

**Conclusions:**

Extraintesinal amoebiasis can be resolved if it is diagnosed early and treated with metronidazole. Clinicians should not neglect amoebiasis in patients presenting with jaundice and right upper quadrant pain.

## 1. Background

*Entamoeba histolytica* is a protozoan that causes intestinal and extraintestinal amoebiasis. In an endemic area, 90% of *E. histolytica* infections are limited to the lumen of the intestine and are asymptomatic. However, gastroenterology reports showed that nearly 50 million people become symptomatic with about 100,000 deaths yearly in the world [1]. Amoebic infections are more prevalent in countries with lower socioeconomic status and poor public health due to ingestion of amoebic cysts through fecal-oral contact, usually through contaminated food or water resources. Trophozoites can invade the intestinal mucosa or blood vessels, reaching extraintestinal sites such as the liver, brain, and lungs [2–4].

Even though the majority of infections restricted to the lumen of the intestine (“luminal amoebiasis”) are asymptomatic, amoebic colitis, or invasive intestinal amoebiasis, occurs when the mucosa is invaded. Trophozoites of *E. histolytica* kill epithelial cells and invade colonic epithelium, progressing to the submucosa. Necrosis progresses laterally and downwards forming “flask-shaped” necrotic tissue which leads to different organ damage and causes secondary bacterial infections. The report indicated that extraintestinal amoebiasis is 3 times more likely to affect middle-aged men between the ages of 18 and 50 [4].

Symptoms include severe dysentery and associated complications. Severe prolonged infections may lead to complications such as perforations, peritonitis, and the formation of amoebic granulomas (amoeboma). Amoebic liver abscesses are the most common form of extraintestinal amoebiasis [5]. We report a patient who initially delivered with intrauterine fetal death (IUFD) after the 9th month of amenorrhea and treated for intestinal amoebiasis with tinidazole but was later found to have extraintestinal amoebiasis related to pregnancy.

## 2. Case presentation

Our client was a 31-year-old female who was on the 6^th^ postpartum day after intrauterine fetal death (IUFD). She claimed to be amenorrheic for nine months, and delivery was at the hospital by spontaneous vaginal delivery. There was a nuchal cord on the freshly dead fetus. On February 28, 2021, she was admitted to Bichena Primary Hospital with severe epigastric pain, hiccups, and bloody vomiting of ingested matter of 5 days. She had also right upper quadrat abdominal pain and fatigue. In addition, she had yellowish discoloration of the eyes for one week. Moreover, she had pruritus, fever, and had a history of watery diarrhea six days ago which got subsided during the presentation.

On physical examination, the patient was acutely sick seeming with pain. Vital signs: blood pressure was 150/100 mmHg, pulse rate was 92 bpm, respiration rate was 32 breaths/min, temperature was 36.6°C, and oxygen saturation was 96% with atmospheric air. On head and neck evaluation, she had icteric sclera and pink conjunctiva. On abdominal examination, she had tenderness at the right upper quadrant of the abdomen. Otherwise, there was no finding in the remaining systems.

Her laboratory investigations revealed serum total bilirubin of 9.4 mg/dL, direct bilirubin of 3.55 mg/dL, alkaline phosphatase of 213 *µ*/L, aspartate transaminase of 42 *µ*/L, alanine transaminase of 40 *µ*/L, creatinine of 0.6 mg/dl, and blood urea nitrogen of 16 mg/dl. She had also bloody urine (blood 3+), no protein and ketone on urine analysis, and trophozoites of *E. histolotica/dispar* were seen in stool microscopically. Her total white blood count was 13000 cell/mm^3^ (neutrophils cover 53.1%; lymphocytes, 10.4%; eosinophils, 2.8%; basophils, 19.6%; and monocytes, 14.1%); red blood cells, 3,700,000 cells/*µ*; Hgb, 12 mg/dl; Hct, 32%; and platelets, 79,000cell/mm^3^. She was negative for hepatitis viral markers and *Helicobacter pylori* in stools.

A diagnosis of obstructive jaundice secondary to choledocholithiasis with ascending cholangitis and intestinal amoebiasis was made, and the patient kept nothing per mouth (NPO). She was admitted and started management with diclofenac 75 mg intramuscularly as needed, cimetidine 400 mg loading intravenous, then 200 twice a day, ceftriaxone 1 gm intravenous twice a day, metrindazone 500 mg intravenous three times a day, nifedipine 20 mg PO twice a day and maintenance fluid with normal saline, ringers lactate, and 5% dextrose as required. On the second day of admission, abdominal ultrasound was done and showed a normal biliary system and no stone at the common bile duct. But, there were multiple lesions on the right lobe of liver parenchyma which were most likely small multiple amebic live abscesses. She was treated onsite with metronidazole 500 mg TID and ceftriaxone 500 mg QID for five days considering extraintestinal amoebiasis that is amoebic liver abscess. Then, she was discharged with metronidazole 500 mg PO three times a day and cloxacillin 500 mg PO four times a day for seven days and appointed for two weeks later.

After two weeks of discharge, a patient come back to the hospital with normal vital signs and tested for hematological profile, liver function, stool sample, and abdominal ultrasound. Her total white blood cell count was 11,000 cell/mm^3^ (neutrophils (56%), lymphocytes (24%), eosinophils (2.3%), basophils (8%), and monocytes (9%)). Red blood cell count was 4.3 million cells/mm^3^; Hgb, 13 mg/dl; Hct, 38%; and platelets, 156,000 cells/mm^3^. Total bilirubin was 3.6 mg/dL; direct bilirubin, 1.2 mg/dL; alkaline phosphatase, 135 *µ*/L; aspartate transaminase, 52 *µ*/L; alanine transaminase 45 *µ*/L; creatinine, 0.5 mg/dl; and blood urea nitrogen, 14 mg/dL. From her stool sample, no ova or parasite is seen and few pus cells were observed. Abdominal ultrasound confirmed absence of lesions on the right lobe of liver parenchyma. We were unable to show the endoscopic images of this patient because of absence of endoscopy access.

## 3. Discussion

Extraintestinal amoebiasis is more likely to occur in the developing world. It especially affects pregnant women on corticosteroid treatment, malignancy, malnutrition, and alcoholic individual. However, we can easily manage this disease if we have appropriate anticipation and correct diagnosis. The reasons for misdiagnosis and late presentation of the disease are not only limited to illiteracy, poverty, lack of awareness, and lack of social support but also because we have limited diagnostic methods and poor exposure of clinicians for the extraintestinal amoebiasis. In developing countries, infection with *E. histolytica* is usually neglected and marginalized [6].

Our patient presented after the 6^th^ day of IUFD delivery and was diagnosed to have amoebic liver abscess. She was admitted for 5 days and treated with metronidazole three times per day intravenous together with antibiotics. A study done in England reported that extraintestinal amoebiasis has a strong association with pregnancy [4]. Trophozoites of *E. histolotica* adhere to colonic epithelial cells through a specific parasite protein: galactose-N-acetyl galactosamine lectin, amebapore, and proteases. Invasion is mediated by the killing of epithelial cells, neutrophils, and lymphocytes by trophozoites, and the colonic epithelial cells were killed through cytolysis and apoptosis, as consequences of interleukin-1*α* and precursor interleukin-1 release. Those cells also produce cytokines and other inflammatory mediators such as COX-2, interleukin-1, interleukin-8, INF *α*, and others. These cytokines and inflammatory mediators subsequently attract neutrophils and macrophages, and this state aggravates mucosal inflammation, thickening, ulcers, and necrosis which can subsequently lead to perforation [4].

In addition, Gal/GalNAc-specific lectin has antigenic cross-reactivity to CD59, a human leukocyte antigen that prevents the assembly of the complement C5b–C9 membrane attack complex. Amoebic cysteine proteinases rapidly degrade the complement anaphylatoxins C3a and C5a [7]. The cysteine proteinases also degrade secretory IgA and serum IgG, possibly protecting amoebae from opsonization [8]. Finally, amoebae appear to suppress both the macrophage respiratory burst and antigen presentation by class II major-histocompatibility complex (MHC) molecules that probably result in fetal distress and cause fetal death.

Our patient had antenatal care follow-up (ANC), and there was no danger sign, there was no history of abortion, and she has an O blood group and RH is positive. She was also dewormed by mebendazole and took iron foliate for two months. She had seen by ultrasound during ANC follow-up, the fetus was actively mobile, and the fetal heartbeat was normal. The amniotic fluid and placenta were good at 6 months of gestational age. Generally, she did not have any known illness. Probably, extraintestinal amoebiasis infection might have triggered toxic substance release (probably vesicles) and inflammatory cells to pass to the fetus through the placenta that caused the IUFD.

Our patient presented with yellowish discoloration of eyes with severe epigastric pain, hiccups, and bloody vomiting of ingested matter of 5 days. She had also right upper quadrant abdominal pain and fatigue. She also presented with yellowish discoloration of eyes. Reports indicated that gastrointestinal symptoms occur in 10 to 35 percent of patients, which include nausea, vomiting, abdominal cramps, abdominal distention, diarrhea, and constipation. Hepatomegaly with point tenderness over the liver, below the ribs, or in the intercostal spaces is a typical finding [9, 10]. Our patient had right upper quadrant abdominal tenderness and icteric sclera. The study showed that less than 10 percent of patients have jaundice [11]. After the patient has discharged, the yellowish discoloration of the eyes decreased, and there was no body swelling.

Laboratory investigation of our patient showed leukocytosis and an increased level of alkaline phosphatase and direct and total bilirubin. Common laboratory findings include leukocytosis without eosinophilia, elevated alkaline phosphatase, transaminitis, and elevated erythrocyte sedimentation rate [10]. In addition, actively motile trophozoites were visualized with an Olympus microscope from a stool sample.

Finally, our patient was treated with diclofenac 75 mg intramuscularly as needed, cimetidine 400 mg intravenous state, then 200 two times a day, ceftriaxone 1 gm intravenous two times a day, and metronidazole 500 mg intravenous three times a day. The study indicated that patients with fulminant amebic colitis will additionally require fluid resuscitation, broad-spectrum antimicrobial therapy for peritonitis, intensive supportive care, and surgical intervention for bowel perforation and bowel necrosis. However, our patient did not have a sign of peritonitis. Metronidazole is highly effective in eliminating invading trophozoites and remains the recommended therapy for amebic colitis and amoebic liver disease. Tinidazole has a longer half-life and is better tolerated, but metronidazole is as effective at clearing parasites [12].

## 4. Conclusions

Amoebiasis is a common parasitic infection, especially in developing countries. Immunocompromised individuals are at a risk of extraintestinal amoebiasis and its complication. Health workers could give attention and open communication especially in obtaining a good history will be critical in early diagnosis and treatment.

## Data Availability

All data generated during this study are included in this article.

## Abbreviations

ANC: Antenatal care
C: Complement
CD: Cluster of differentiation
*E. histolytica*: *Entamoeba histolytica*
Gal/GalNAc: Galactose-N-acetyl galactosamine
IL: Interleukin
Im: Intramuscle
IUFD: Intrauterine fetal death
Iv: Intravenous.
